# Combining glucose and sodium acetate improves the growth of *Neochloris oleoabundans* under mixotrophic conditions

**DOI:** 10.1186/s13568-016-0180-5

**Published:** 2016-02-05

**Authors:** Helder Rodrigues Silva, Cassio Egidio Cavenaghi Prete, Freddy Zambrano, Victor Hugo de Mello, Cesar Augusto Tischer, Diva Souza Andrade

**Affiliations:** Dept of Agronomy, Universidade Estadual de Londrina, Londrina, Paraná 86051900 Brazil; Dept of Biotechnology, Universidade Estadual de Londrina, Londrina, Paraná 86051990 Brazil; College of Chemistry, Universidade Norte do Paraná, Londrina, Paraná 86041120 Brazil; Instituto Agronômico do Paraná, CP 480, Londrina, Paraná 86.047-902 Brazil

**Keywords:** BBM dilution, Free fatty acid, Mixotrophic, Total lipid, Total protein, Response surface methodology

## Abstract

Mixotrophic cultivation is a potential approach to produce microalgal biomass that can be used as raw materials for renewable biofuels and animal feed, although using a suitable, cost-effective organic carbon source is crucial. Here, we used a Box–Behnken design with three factors, the glucose and sodium acetate concentrations, and the percentage of Bold’s basal medium (BBM), to evaluate the effects of different carbon sources on biomass productivity and the protein and lipid contents of *Neochloris oleoabundans* (UTEX#1185). When grow at optimal levels of these factors, 100 % BBM plus 7.5 g L^−1^ each of glucose and sodium acetate, *N. oleoabundans* yielded 1.75 g L^−1^ of dry biomass, with 4.88 ± 0.09 % N, 24.01 ± 0.29–30.5 ± 0.38 % protein, and 34.4 % ± 0.81 lipids. A nuclear magnetic resonance spectrum (^1^H-NMR) of a lipid extract showed that the free fatty acid content was 11.25 %. Thus, combining glucose and sodium acetate during the mixotrophic cultivation of *N. oleoabundans* can yield greater amounts of biomass, proteins, and lipids for biofuel production.

## Introduction

Increasing use of oil-derived energy has generated negative environmental effects such as pollution and greenhouse gas emissions. To seek alternatives sources of bioenergy, several studies of the growth of photosynthetic microorganisms suggested that microalgae can be used to produce sustainable feedstocks for energy production (Amaro et al. 2011; Georgianna and Mayfield 2012; Ghosh et al. 2015; Pruvost et al. 2011; Zhang et al. 2011), including bioethanol via enzymatic hydrolysis (Kim et al. 2014) and biogas, such as biohydrogen and biomethane, from anaerobic fermentation (Wieczorek et al. 2014). Extracted oils from the biomass of some genera of microalgae have a high free fatty acid (FFA) content that can account for up to 85 % of the total lipids (Chen et al. 2012; Krohn et al. 2011). Microalgal biomass storage conditions are very important factors, as they can decrease the lipid content, release organic volatile acids, and/or lead to the formation of FFAs (Alencar et al. 2010; Foree and McCarty 1970). In this regard, Chen et al. (2012) indicated that when biomass was stored at temperatures above the freezing point, the lipids, such as triacylglycerol, in microalgae can be hydrolyzed to FFAs by lipases, peroxidases, and phospholipases that are present in wet paste or contaminating microorganisms.

In addition to their great potential for bioenergy production, some species of microalgae are already used in aquaculture, the production of food supplements, and the pharmaceutical industry to extract high-value compounds (Gatenby et al. 2003), as well as in bioremediation and biofertilization. Biomolecules can be obtained as byproducts from microalgae during biofuel production, and their subsequent utilization in animal and aquaculture feed might sustain an industrial-scale cultivation system (Yaakob et al. 2014). A mixotrophic system is defined as one in which organic carbon sources, such as molasses, glucose, glycerol, sucrose, lactose, starch, and CO_2_, are simultaneously assimilated by respiratory and photosynthetic metabolic pathways (Alkhamis and Qin 2015; Rai et al. 2013; Yeesang and Cheirsilp 2014). A mixotrophic system combines the advantages of phototrophic and heterotrophic cultures (Li et al. 2014), and obtains energy from organic and inorganic carbon sources, as well as light. In mixotrophic cultivation, microalgal cultures produce more biomass (Babuskin et al. 2014), including higher amounts of pigments and fatty acids, than phototrophic systems (Alkhamis and Qin 2015).

It is believed that improved biomass productivity because of mixotrophy also enhances lipid and protein yields, at least for species such as *Nannochloropsis oculata*, *Dunaliella salina*, and *Chlorella sorokiniana* (Wan et al. 2011). Glucose is the final product of photosynthesis, and it is assumed that photosynthetic microalgae must be able to metabolize it. Thus, it is reasonable to expect that glucose metabolism is straightforward (Garcia et al. 2005). Improved culture media that contain different concentrations of glucose or sodium acetate as carbon sources have been analyzed extensively for microalgae cultivation (Estévez-Landazábal et al. 2013; Huang et al. 2010; Jeon et al. 2006; Li et al. 2014; Moon et al. 2013; Rai et al. 2013; Wang et al. 2012; Zhang et al. 2011). However, to our knowledge, no published study has used a response surface methodology to assess the effects of these two carbon sources on microalgal growth.

The objective of this study was to evaluate a *Neochloris oleoabundans* strain under mixotrophic growth conditions by combining glucose and sodium acetate as carbon sources, with a focus on the production and characterization of microalgal biomass and lipids. Here, we report the rate and yield of biomass production by *N. oleoabundans* after 10 and 15 days of cultivation, and how they are influenced by combining various percentages of Bold’s basal medium (BBM) and glucose and sodium acetate concentrations. We used a novel response surface analysis to examine the best combination of these factors to optimize the cell density and dry biomass production of *N. oleoabundans*. We also show that in a vertical photobioreactor that uses sunlight, the microalgal biomass has high lipid and protein contents, but its lipid profile has few FFAs.

## Materials and methods

### Microalgae and growth medium conditions

The *N. oleoabundans* UTEX#1185 strain was purchased from the Culture Collection of Algae at the University of Texas at Austin, Austin, TX, USA, and kept in axenic liquid BBM at the Microbial Collection (IPR) of the Instituto Agronomic Institute of Paraná (IAPAR) Paraná, Brazil. Two experiments were conducted: one in the laboratory and one in an outdoor tubular photobioreactor at experimental station of the IAPAR, in the municipality of Londrina in North Paraná State, Brazil (lat. 23°08′47″S, long. 51°19′1″W, 640 m a.s.l.).

For all experiments, microalgae were grown in axenic liquid BBM containing the following reagents (g L^−1^): NaNO_3_, 0.25; CaCl_2_·2H_2_O, 0.025; MgSO_4_·7H_2_O, 0.075; K_2_HPO_4_, 0.075; KH_2_PO_4_, 0.175; NaCl, 0.025; C_10_H_12_N_2_Na_4_O_8_, 0.0498; ferric solution (FeSO_4_·7H_2_O, 0.00498; and 1 mL^−L^ of H_2_SO_4_). The following reagents were also used (mg L^−1^): H_3_BO_3_, 11.42; ZnSO_4_·7H_2_O, 1.41; MnCl_2_·4H_2_O, 1.44; CuSO_4_·5H_2_O, 1.57; NaMoO_4_·5H_2_O, 0.192; and Co(NO_3_)_2_·6H_2_O, 0.045 (Bold 1949). The BBM was modified by the addition of different concentrations of glucose (C_6_H_12_O_6_) and sodium acetate (CH_3_COONa) as carbon sources. The pH of the BBM was adjusted to 9.0 with 0.1 M KOH and sterilized by autoclaving at 121 °C and 1.5 atm for 30 min.

### Experimental design and treatments

The first experiment was performed with a Box–Behnken design using STATISTICA software v7.0 (Statsoft 2007), and the results were analyzed by a response surface methodology for three independent variables (the sodium acetate and glucose concentrations and the dilution ratio in BBM, which was expressed as % BBM). The factor levels and the independent variables are presented in Table 1. In the first part of the study, 13 trials were performed with three replicates as follows: three encoded levels (−1, 0, 1) in 13 trials and three factors (A, B, C, which correspond to the concentrations of glucose and sodium acetate, and the % BBM, respectively), were analyzed (Table 2).

**Table 1:** Level of factors, carbon sources (glucose and sodium acetate) concentrations in g L^−1^, the dilution of medium (Bold’s Basal Medium, BBM) in percentage (%) and the corresponding encoded levels (X) in the Box-Behnken design

	Range of levels (X)		
Factors	−1	0	1
A (Glucose in g L^−1^)	0	5	10
B (Sodium acetate in g L^−1^)	0	5	10
C (BBM in %)	50	75	100

**Table 2:** Experimental design *Box*-*Behnken* for three independent variables and experimental data with 13 trials

Run	Independent variables			Dependent variables			
	Glucose	Sodium acetate g L^−1^	BBM (%)	OD_670_		Dry biomass g L^−1^	
				10 day	15 day	10 day	15 day
1	−1 (0.0)	−1 (0.0)	0 (75)	0.170	0.100	0.080	0.100
2	1 (10.0)	−1 (0.0)	0 (75)	0.750	0.826	0.725	0.485
3	−1 (0.0)	1 (10.0)	0 (75)	0.203	0.215	0.264	0.189
4	1 (10.0)	1 (10.0)	0 (75)	1.422	1.619	0.921	1.233
5	−1 (0.0)	0 (5.0)	−1 (50)	0.305	0.260	0.150	0.180
6	1 (10.0)	0 (10.0)	1 (50)	1.549	1.555	0.962	1.029
7	−1 (0.0)	0 (5.0)	1 (100)	0.340	0.304	0.240	0.200
8	1 (10.0)	0 (5.0)	1 (100)	1.729	1.452	1.195	1.048
9	0 (5.0)	−1 (0.0)	−1 (50)	0.726	0.699	0.436	0.456
10	0 (5.0)	1 (10.0)	−1 (50)	1.423	1.504	0.911	0.989
11	0 (5.0)	−1 (0.0)	1 (100)	1.133	1.047	0.762	0.636
12	0 (5.0)	1 (10.0)	1 (100)	1.807	1.378	1.529	0.971
13	0 (5.0)	0 (5.0)	0 (75)	1.677	1.649	1.223	1.059

The optical density at 670 nm (OD_670_) and dry biomass determinations *N. Oleoabundans* were performed after 10 and 15 days of cultivation

### Growth chamber experiment

A microalgal inoculum was prepared by growing cells in clear glass tubes containing 100 mL of sterilized BBM at an initial pH of 9.0. Media that contained supplementary carbon sources according to the treatments (Table 2) were inoculated with 10 % (v/v) of a culture of a green microalgae *N.oleoabundans* strain with a density of 1.0 × 10^6^ cells mL^−1^. The assays were conducted in a growth chamber with a 12 h:12 h light:dark photoperiod at 28.0 ± 2.0 °C in the light phase and 22.0 ± 2.0 °C in the dark phase. Illumination in the growth chamber was provided by white, cool fluorescent lamps in the form of tubes that were arranged in parallel with the upper part of the cultivation container. The photon flux density of photosynthetically active radiation was 100 ± 20 µE m^−2^ s^−1^, which was measured at the surface of the flasks using a liquor porometer (INC model LI-1600).

### Optical density and biomass determinations

The optical density at 670 nm (OD_670_) and dry biomass determinations were performed after 10 and 15 days of cultivation. The OD_670_ was determined using a Genesys 10 UV spectrophotometer. To determine the dry biomass, a 40-mL aliquot was collected from each flask and centrifuged (Model Z383 HERMLE K) at 11,536 g for 10 min at 25.0 °C. The pellet was dried to a constant weight in an oven at 60 °C.

### Photobioreactor growth conditions

After analyzing the results of the first experiment using a response surface methodology, a second experiment was performed in closed, vertical, tubular photobioreactors that were constructed with low-density polyethylene and which contained 20 L of medium with air injection (Silva et al. 2014). The culture medium consisted of 100 % BBM supplemented with 7.5 g L^−1^ glucose and 7.5 g L^−1^ sodium acetate. The cultivation was conducted in October 2014 in triplicate, and the pH of the medium was maintained at 9.0 using 0.1 M KOH. The closed, vertical photobioreactors were kept outdoors, and during the cultivation, temperature and solar radiation (W/m^2^) data were obtained from nearby meteorological stations (IAPAR 2014; Paraná 2014).

### Growth and biomass determinations

A 50-mL aliquot was collected daily from the photobioreactor for the OD_670_ and dry biomass analyses that were performed as described in the growth chamber experiment. On day 10 of cultivation, the cell density was determined by counting cells with an improved Neubauer hemocytometer using an optical microscope (Eclipse E200, Nikon) with a 40× objective and a visual magnification of 400×. Biomass productivity in mg L^−1^ d^−1^ was calculated from the variations in biomass concentration (in mg L^−1^) at different cultivation times (in d) according to the following equation:

1 $$p = x_{ 1} - x_{0} /t_{ 1} - t_{0}$$

where *x*_1_ and *x*_0_ are the biomass concentrations (in mg L^−1^) on d *t*_1_ and *t*_0,_ respectively.

The specific growth rate (µ d^−1^) was calculated as described previously (Kong et al. 2013; Li et al. 2014) using the following equation:

2 $$\mu = \, \left( {{ \ln }x_{ 1} - { \ln }x_{0} } \right)/t_{ 1} - t_{0}$$

where *x*_1_ and *x*_0_ are the biomass concentrations (in g L^−1^) on d *t*_1_ and *t*_0_, respectively.

### Lipid and protein contents

On day 10 of cultivation, the biomass was harvested by concentrating the entire volume (20 L) by centrifugation in 250-mL bottles at 25.0 °C at 2336 g for 20 min. Then, the supernatant was transferred to Falcon tubes that were centrifuged at 11,536 g for 10 min. Subsequently, the biomass was freeze-dried in a lyophilizer (LIOBRAZ L101 model) for lipid and protein content determinations. Total lipids were extracted from 100 mg of lyophilized microalgal biomass using the method described by Bligh and Dyer (1959) and the procedure given by Ryckebosch et al. (2012). The lipids were extracted with chloroform, methanol, and water at a ratio 1:1:0.8. Total nitrogen (N) was determined by the Kjeldahl micro-method using 100 mg of lyophilized biomass (Bremner 1965). Total protein contents were calculated using the total N data and conversion factors of 6.25, according to Alkhamis and Qin (2015), and 4.92, as recommend for green, brown, and red marine algae by Lourenço et al. (2002). All assays were conducted in triplicate.

### Nuclear magnetic resonance (NMR) analysis of FFAs

The extract to lipid analysis was obtained with procedure used to determine the total lipid content described by Bligh and Dyer (1959) and modified by Ryckebosch et al. (2012). The lipid profile was obtained by NMR that was performed at the Multiuser Laboratory of Spectroscopy—SPEC-UEL, State University of Londrina, Londrina, Brazil,. The samples were dissolved in CDCl_3_ solvent and analyzed using a Bruker Avance III 400 MHz spectrometer equipped with a 5 mm double resonance broadband inverse (BBI) probe at 303 K. The ^1^H-NMR experiments were performed at 400.13 MHz with the standard pulse sequences described by Braun and collaborators (2000). The FFA degree of total lipid was calculated from the area of the peaks obtained from deconvoluted spectrum using 50/50 Gaussian/Laurentzian; the α-carbonyl methylene hydrogens were counted as six hydrogens, and C1 and C3 -CH_2_-O- as four hydrogens, considering for 100 % esterification (Carneiro et al. 2005).

## Results

### Dry biomass

**Fig. 1:**
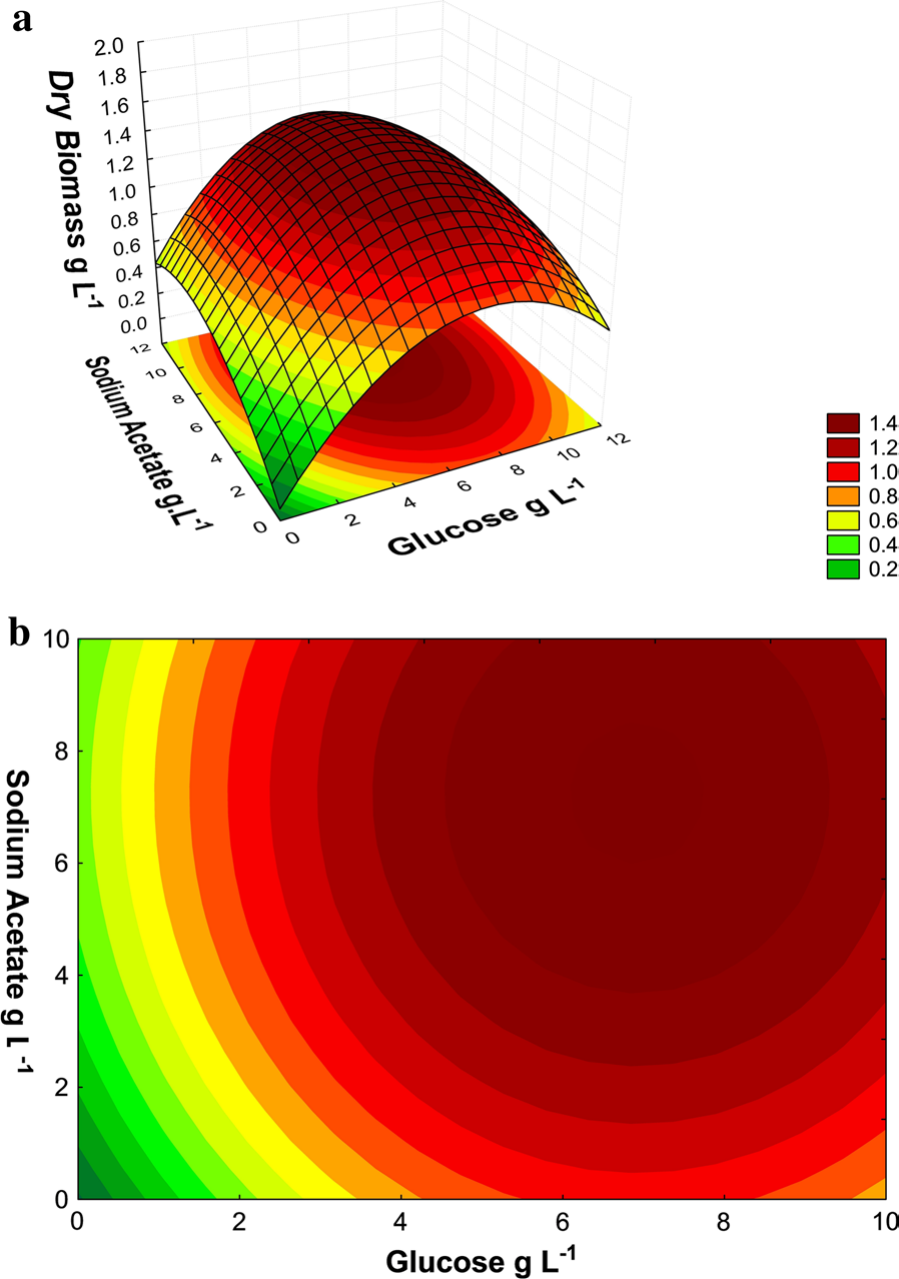
Dry biomass (g L^−1^) of the *N. oleoabundans* on day 10 of cultivation in BBM (100 %). **a** *Response surface* and **b** *curve contour*, showing the interactions between the concentrations (g L^−1^) of the sodium acetate and glucose

In the first experiment, the initial inoculum had a concentration of 1.67 × 10^7^ cells mL^−1^, a dry biomass concentration of 0.29 g L^−1^, an OD_670_ of 0.52, and a pH of 8.70. The variables studied had significant (*p* < 0.01) effects on the dry biomass of *N. oleoabundans*, and the highest value was 1.4 g L^−1^, which was obtained on day 10 of cultivation (Fig. 1a, b). The regression coefficient (R^2^ = 0.97) showed that 97 % of the variability could be explained by the model and the best response prediction (Burkert et al. 2004; Safaralie et al. 2010). To confirm the validity and the model fit, an assessment of the experimental data was performed using analysis of variance, and the model presented a value of *p* = 0.20 for the lack of fit, which is not significant, indicating that the model can be used for predictive purposes (Tovar et al. 2010). The response surface and contour curve (Fig. 1a, b) were obtained from the regression coefficient after analysis of the fitted model, as shown in the following equation:

3 $$z = \, - 1. 2 5 4 { } + \, 0. 2 6 6x - \, 0.0 1 7x^{ 2} + \, 0.0 9 7y - \, 0.00 9y^{ 2} + { 1}.0 5\times 10^{ 4} \times \, \left( {xy^{ 2} } \right) \, - { 3}. 3 2\times 10^{ - 4} \times \, \left( {x^{ 2} y} \right) \, - { 2}.0 5\times 10^{ - 6} \times \, \left( { 100x^{ 2} } \right)^{{}} + { 5}. 7 8\times 10^{ - 4} \times \, \left( { 100y} \right) \, + { 1}. 1 3 5$$

The analysis of the means of dry biomass production (g L^−1^) assayed on d 10 of cultivation was influenced by a combination of the concentrations of glucose and sodium acetate in the medium, as well as by the % BBM (Fig. 1a). On day 15 of cultivation, the regression coefficient obtained was R^2^ = 0.98. The analysis of variance indicated that the lack of fit (*p* = 0.15) was not significant, confirming the validity of the model. From the regression coefficient and model fit analysis, the response surface and contour curve were obtained as shown in Eq. (4):

4 $$z = - 1.00 6 { } + \, 0. 1 5 7x - 0.00 6x^{ 2} + \, 0.0 8 9y - 0.00 6y^{ 2} + \, 0.00 5xy + \, \left( { 4. 6 5 6\times 10^{ - 6} \times 10,\!000x} \right) - \left( { 8. 8 4 1\times 10^{ - 5} \times { 1}00x^{ 2} } \right)^{{}} - \left( { 1. 4 90 \, \times { 1}0^{ - 6} \times 10,\!000y} \right) \, + { 1}.0 1 4$$

**Fig. 2:**
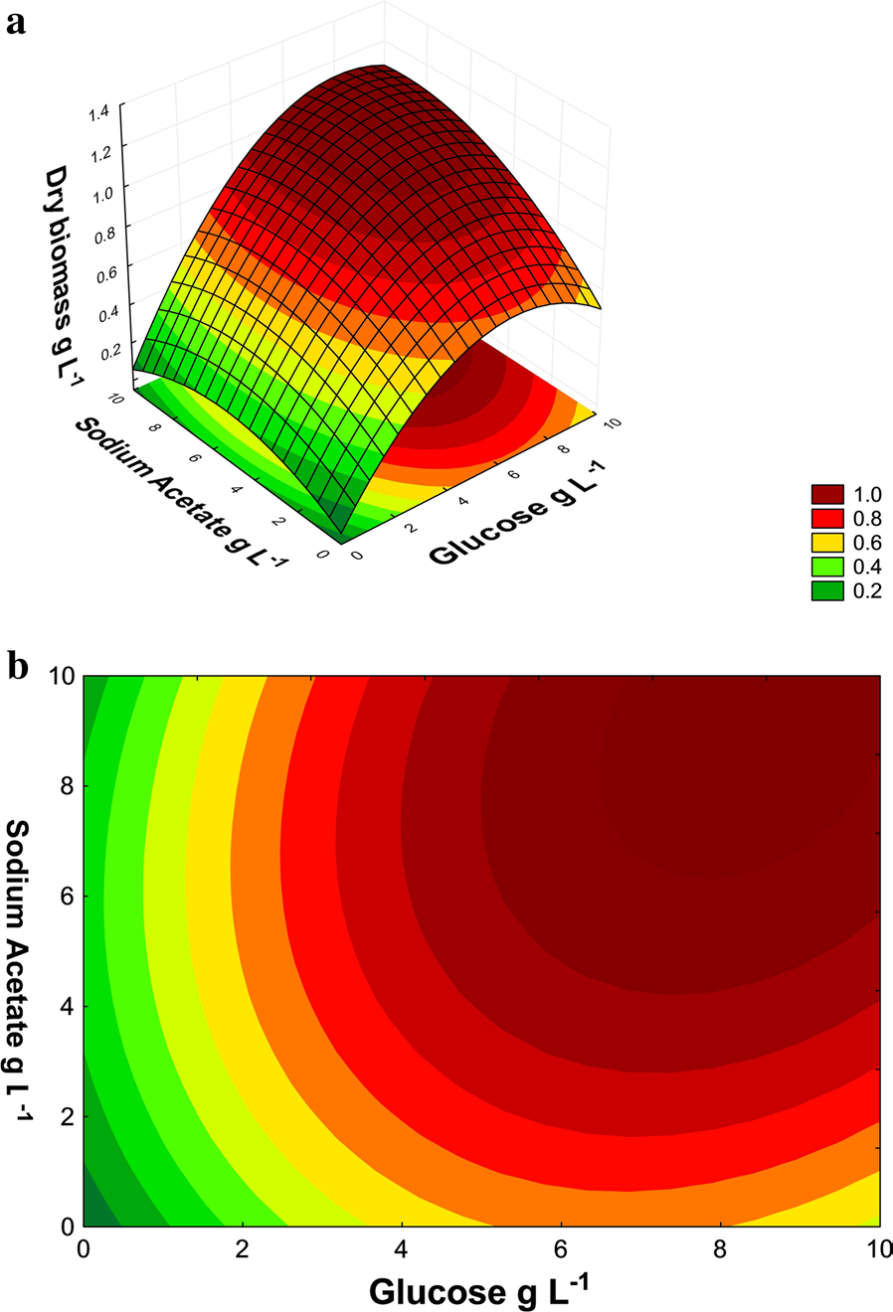
Dry biomass (g L^−1^) of the *N. oleoabundans* on day 15 of cultivation in BBM (100 %). **a** *Response surface* and **b** *curve contour*, showing the interactions between the concentrations (g L^−1^) of the sodium acetate and glucose

In the response surface study, the % BBM variable was kept at 100 %, and the results obtained were similar to those on day 10 of the cultivation (Fig. 2a, b). Thus, the microalgal biomass was influenced by different concentrations of glucose and sodium acetate.

### OD_670_ analysis

OD_670_ analyses of microalgal cultures were performed on d 10 and 15. On d 10, the obtained regression coefficient was R^2^ = 0.99, and the lack of fit of the experimental data was verified by analysis of variance. The model presented a value of *p* = 0.55, which is not significant, thus confirming the validity of the model. The response surface and contour curve are represented by the following equation:

5 $$z = \, - 0. 1 5 4 { } + \, 0.0 6 3x - \, 0.00 2x^{ 2} + \, 0.0 6 9y - \, 0.00 6y^{ 2} + \, 0.0 3 6xy - \, 0.00 1xy^{ 2} - \, 0.00 1x^{ 2} y + \, 0.00 2 { } \times { 87}. 5x - \, \left( { 2. 30. 10^{ - 4} \times { 87}. 5x^{ 2} } \right)^{{}} + \, 0. 3 2 5 5$$

**Fig. 3:**
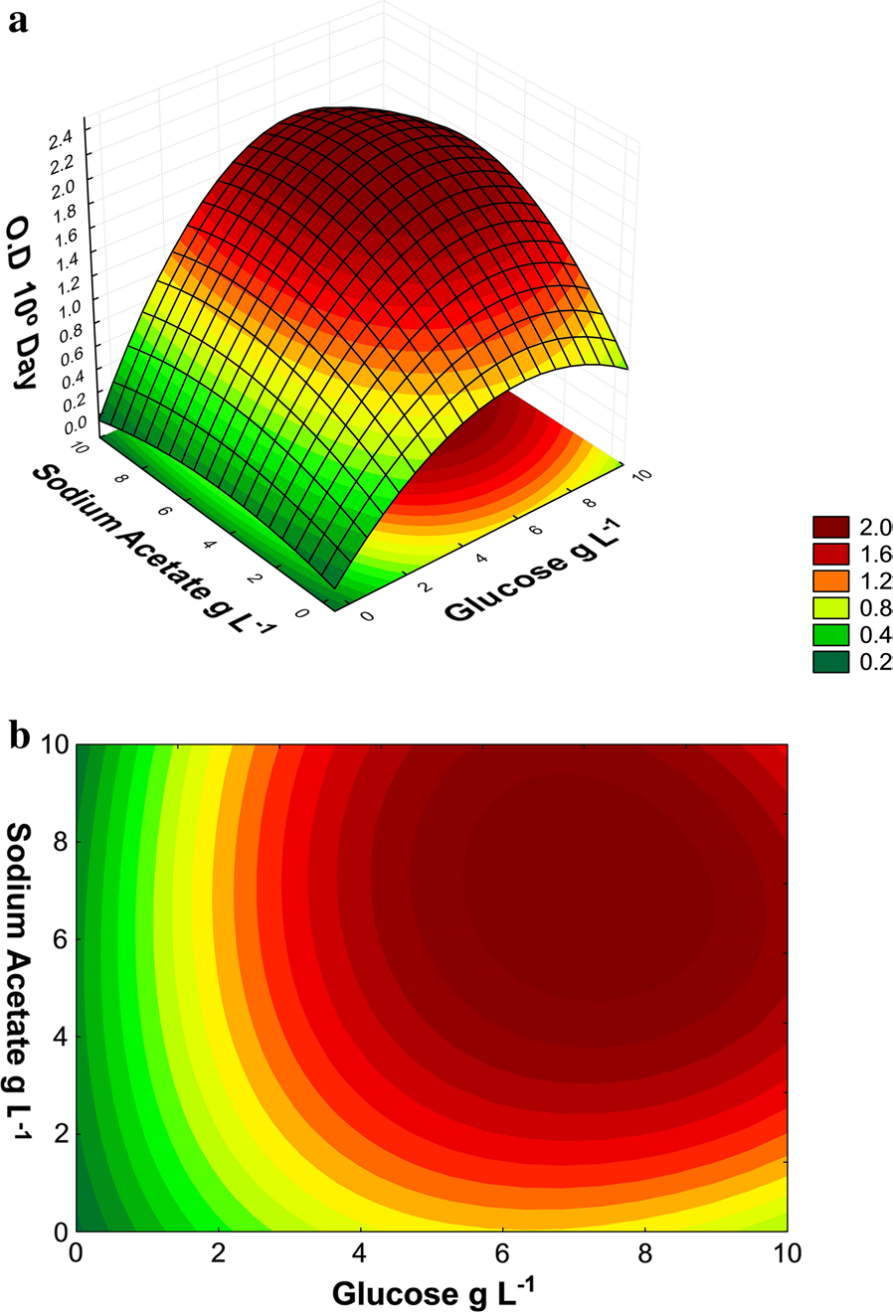
Optical density (OD_670_) of the *N. oleoabundans* on day 10 of cultivation in BBM (100 %). **a** *Response surface* and **b** *curve contour*, showing the interactions between the concentrations (g L^−1^) of the sodium acetate and glucose

An analysis of means based on the concentrations of glucose and sodium acetate in 100 % BBM showed that the highest OD_670_ (2.0) was obtained when the glucose concentration ranged from 5 to 9 g L^−1^ and the sodium acetate concentration ranged from 4 to 10 g L^−1^ (Fig. 3a, b). These results demonstrate that there is a positive relationship between the OD_670_ and biomass production.

On day 15 of cultivation, the obtained regression coefficient was R^2^ = 0.97, and the lack of fit of the experimental data was analyzed by analysis of variance. The model presented a value of *p* = 0.41, which is not significant, thus confirming the validity of the model. The response surface and contour curve are represented by Eq. (6):

6 $$z = \, - 1. 2 3 3 { } + \, 0. 2 6 4x - \, 0.0 1 9x^{ 2} + \, 0. 2 3 2y - \, 0.0 1 8y^{ 2} + \, 0.0 2 5xy - \, \left( { 8. 5\times 10^{ - 4} \times xy^{ 2} } \right)^{{}} - \, 0.00 1x^{ 2} y - \, 0.00 1 { } \times { 5}0y + \, \left( { 1. 30 8\times 10^{ - 4} \times { 5}0y^{ 2} } \right)^{{}} + { 1}. 1 4 1$$

**Fig. 4:**
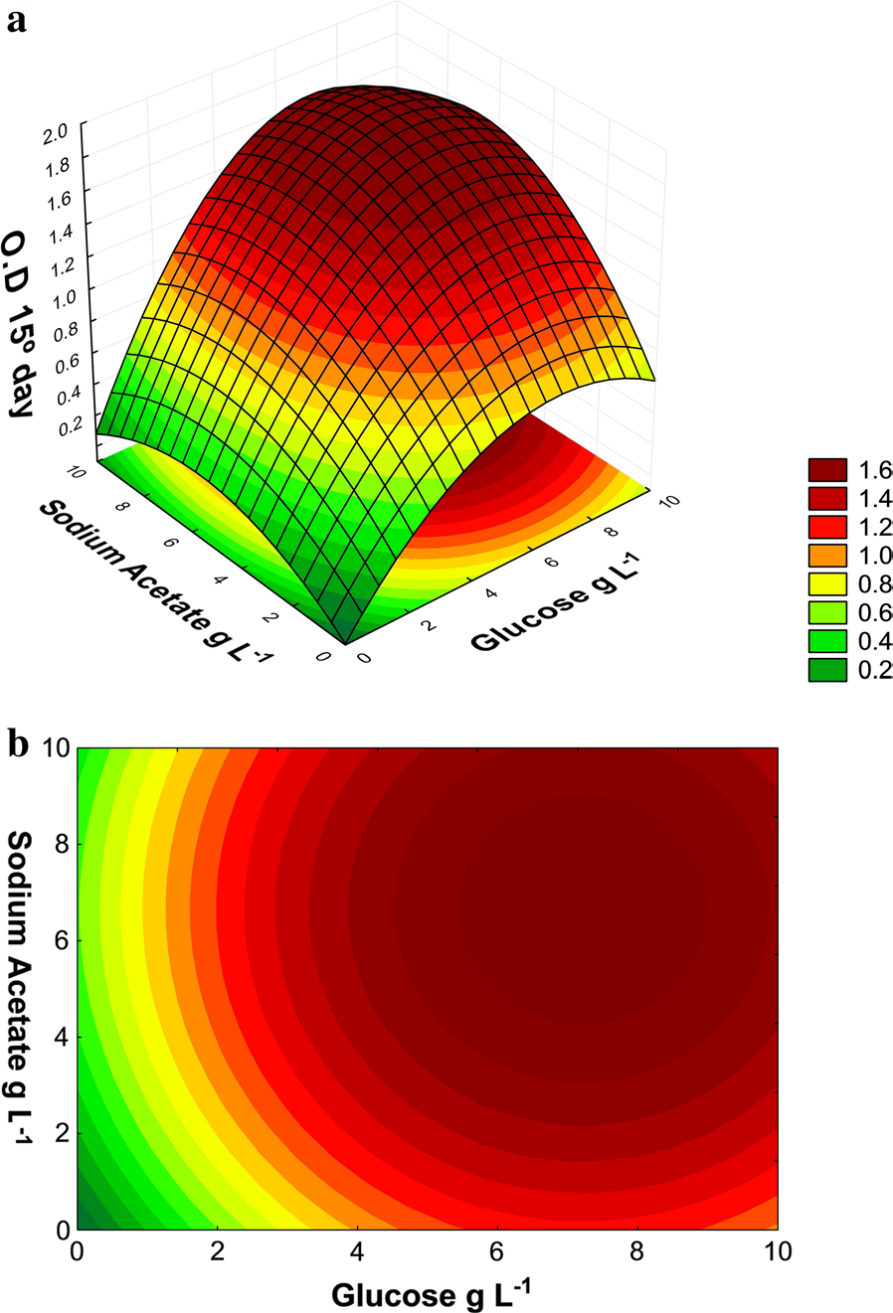
Optical density (OD_670_) of the *N. oleoabundans* on day 15 of cultivation in BBM (100 %). **a** *Response surface* and **b** *contour curve* showing the interactions between the concentrations (g L^−1^) of the acetate sodium and glucose

**Fig. 5:**
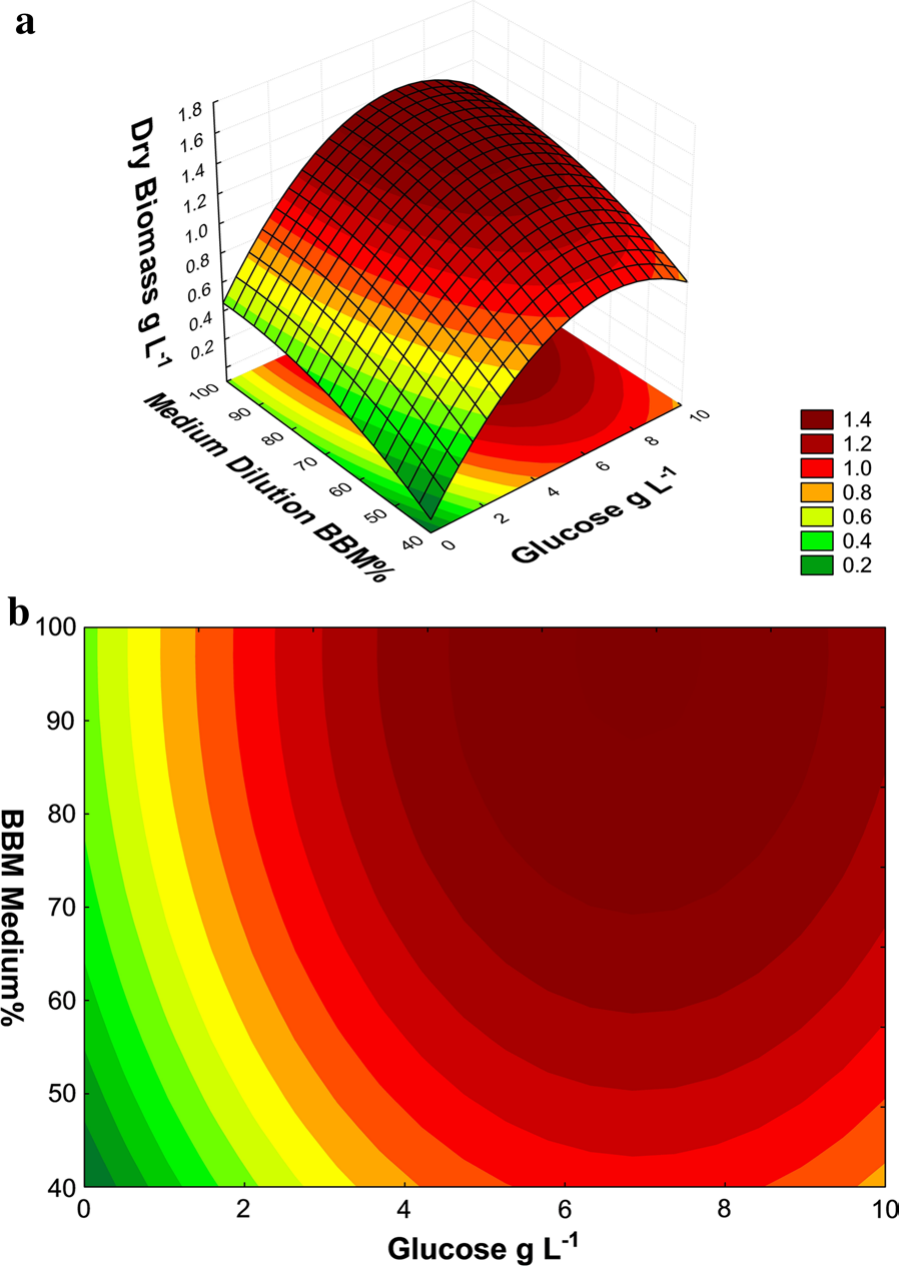
Dry biomass (g L^−1^) of the *N. oleoabundans* on day 10 of cultivation in medium contained the concentration of 7.5 g L^−1^ of sodium acetate. **a** *Response surface* and **b** *contour curve* showing the interactions between the % BBM and glucose concentration (g L^−1^)

The highest average OD_670_, 1.6, was obtained when the glucose concentration ranged from 5.0 to 9.0 g L^−1^ and the sodium acetate concentration ranged from 4.0 to 10.0 g L^−1^ in the BBM (Fig. 4a, b). According to Fig. 5a, b, when working with a concentration of 7.5 g L^−1^ of sodium acetate and glucose, BBM dilution can be done, since above 60 % are obtained an average 1.2 g L^−1^ of dry biomass on d 10 of cultivation. The response surface and contour curve are represented by the following equation:

7 $$z = \, - 1. 3 4 9 { } + \, 0. 2 7 6x - \, 0.0 1 9x^{ 2} + \, 0.0 2 7y - \, \left( { 1. 3 9 5\times 10^{ - 4} \times y^{ 2} } \right)^{{}} + \, 0. 4 7 4$$

### Growth in outdoor photobioreactors

**Fig. 6:**
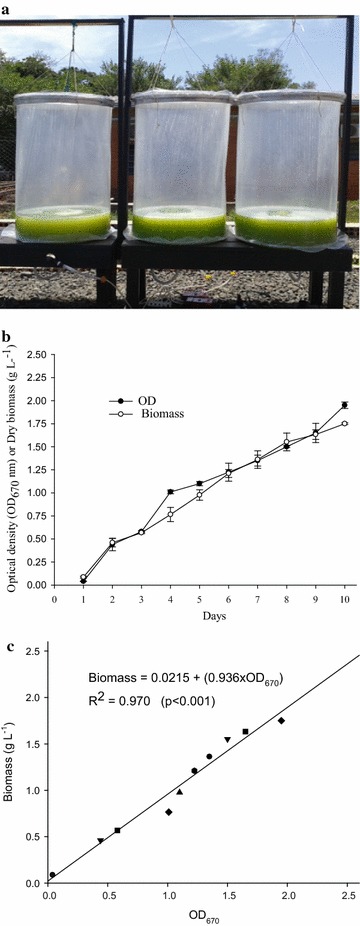
**a** Vertical tubular photobioreactor low density polyethylene with air injection, **b** Growth curve of *N. oleabundans*: absorbance OD_670_ of the culture medium and dry biomass in g L^−1^, **c** linear regression between biomass and optical density (OD_670_). *Bars* represent standard deviations, n = 3

To provide aeration and mixing in each photobioreactor without contaminating the growth media, air was flowed through a 0.22-µm filter (Millipore) that was connected to an injection pump (FRISKAM^®^ Super Model II) (Fig. 6a). The air temperature during the 10-day cultivation in the photobioreactor averaged 28.4 °C, with a maximum of 31 °C and a minimum of 21.5 °C (IAPAR 2014). The solar radiation recorded averaged 414.3 W/m^2^, with a maximum of 563 W/m^2^ and a minimum of 92.9 W/m^2^ (Paraná 2014).

The initial inoculum had an OD_670_ of 0.14, a concentration of 9.9 × 10^6^ cells per mL, a pH of 7.6, and 0.03 g L^−1^ of dry biomass. The culture medium was based on the optimal concentrations determined in the first part of the this study, which were 7.5 g L^−1^ of glucose, 7.5 g L^−1^ of sodium acetate, and 100 % BBM. The growth curve based on the cell concentrations showed that there was a linear increase over the time period examined (Fig. 6b). The highest average OD_670_ (1.95 ± 0.03) was observed on day 10 of cultivation.

The means of dry biomass in g L^−1^ and their respective standard deviations during 10 day of cultivation of *N. oleoabundans* in the three photobioreactors are shown in Fig. 6b. The dry biomass production over the 10-day cultivation period linearly increased, with the lowest yield (0.08 ± 0.01 g L^−1^) on d 1 of cultivation. The highest mean dry biomass (1.75 ± 0.01 g L^−1^) occurred on day 10 of cultivation.

Overall, the different measures of *N. oleoabundans* development in the photobioreactors show that growth occurred as expected, because our system conditions only provide sunlight and a low amount of CO_2_ from the air. The number of cells averaged 8.06 × 10^7^ cells mL^−1^. The specific growth rate (µ) was 0.145 d^−1^. There was a linear relationship [g L^−1^ biomass = 0.0215 + (0.936 × OD_670_)] between the OD_670_ and the biomass, in terms of the dry cell weight, with a correlation coefficient of R^2^ = 0.970 (*p* < 0.001), which passed (*p* = 0.388) the Shapiro–Wilk normality test (Fig. 6c).

An analysis of the lyophilized biomass showed that under mixotrophic conditions, the average composition of *N. oleoabundans* biomass was 4.88 ± 0.09 % N, 24.01 ± 0.29–30.5 ± 0.38 % protein, and 34.4 % ± 0.81 lipids (Table 3).

**Table 3:** Cell count (cells mL^−1^), dry biomass production (g L^−1^) and productivity, total nitrogen (N), protein in dry matter, percentage of lipids, and Free fatty acid (FFA) of the *N. Oleoabundans*, growing in an outdoor vertical photobioreactors under mixotrophic conditions on day 10

Characteristics	**Means (SD)**
Cell count (cells mL^−1^)	8.06 × 10^7^ ± 0.11
Dry biomass production (g L^−1^)	1.75 ± 0.01
Dry biomass productivity (mg L^−1^ d^−1^)	184.81 ± 0.05
^a^Total N in biomass (%)	4.88 ± 0.09
^b^Protein in biomass (%)	30.5 ± 0.38
^c^Protein in biomass (%)	24.01 ± 0.29
Lipid in the biomass (%)	34.7 ± 0.81
Free fatty acid (%)	11.25 %

^a^Kjeldal determination; ^b, c^calculated with factors 6.25 and 4.92, respectively. Data are means of three replicates ± standard deviation (SD)

**Fig. 7:**
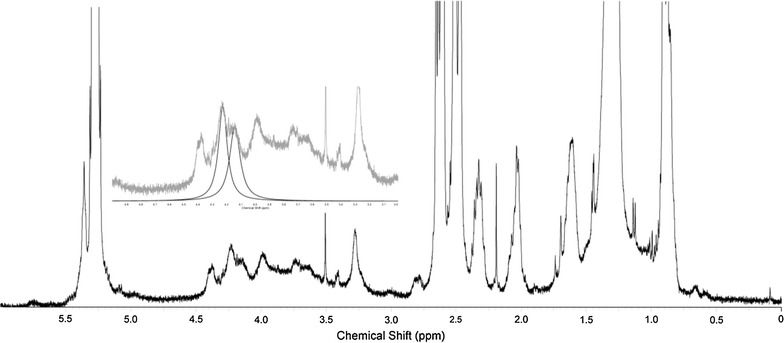
^1^H-NMR spectrum of the lipidic extract of *N. oleoabundans*. On the insert, in *grey line*, is showed the range of 4.10–4.35 ppm corresponding to -CH_2_-O- hydrogens from C1 and C3 of fatty acid-esterified glycerol, as well in *dark line*, the deconvoluted resulted peaks from peak fitting from this region

The NMR spectrum showed clearly all signals expected for triacyglycerol according to Carneiro et al. (2005), and signals between 4.10 and 4.35 ppm that corresponds to -CH_2_-O-, which is typical for C1 and C3 of fatty acid-esterified glycerol (Fig. 7, insert). Regarding the lipid profile of *N. oleoabundans* growing outdoors in a mixotrophic culture medium with optimum glucose and sodium acetate levels, the percent of FFA content was 11.25 % as measured by ^1^H-NMR (Table 3).

## Discussion

*N. oleoabundans* yielded a maximum OD_670_ of 2.0 and a dry biomass concentration of 1.4 g L^−1^ when grown at optimal levels of the factors that were obtained using a response surface methodology analysis (100 % BBM plus 7.5 g L^−1^ each of glucose and sodium acetate). The Chlorophyta *N. oleoabundans* (syn. *Ettlia oleoabundans*) (*Sphaeropleales*, *Neochloridaceae*) strain UTEX#1185, which was isolated from Saudi Arabian sand dunes, is considered to be a halotolerant strain that thrives under phototrophic and heterotrophic conditions. Therefore, this green microalgal strain was chosen for our study because of its strong tolerance to high alkalinity and salinity and, as a oleaginous microalga, it is known to produce high levels of lipids (Gouveia and Oliveira 2009), even when grown under mixotrophic conditions (Baldisserotto et al. 2014).

### Dry biomass production and OD_670_

Increasing the glucose concentration from 5.0 to 9.0 g L^−1^ and the sodium acetate concentration from 4.0 to 10.0 g L^−1^ increased the dry biomass production of *N. oleoabundans* to 1.4 g L^−1^. As there was interaction between these two factors, it was necessary to add both carbon sources to maximize the biomass production. Similarly, Kong et al. (2013) reported that there was a significant interaction between glycerol and glucose on the production and biochemical composition of biomass after *C. vulgaris* was cultivated under mixotrophic conditions for 96 h. Prior to exploring the effects of combinations of glucose and sodium acetate on *N. oleoabundans* growth, we investigated the use of glycerol in mixotrophic cultures, and showed that growth was inhibited after 4 days. This may have resulted from a rapid decrease in the pH of the medium, which resulted in enormous numbers of dead cells.

It was evident from the dry biomass and OD_670_ data that the best results were achieved on day 10, rather than on day 15, of cultivation. Thus, we chose to optimize a 10-day period of cultivation in an outdoor, vertical photobioreactor. On day 10 of cultivation, the optimum concentrations of sodium acetate and glucose for dry biomass production were both 7.5 g L^−1^. With the response surface analysis, we illustrated the similarity between the OD_670_ and the production of biomass (g L^−1^). However, the optimum % BBM varied, as shown in Fig. 5a, b. In our study, the Box–Behnken experimental design was proven to be a good tool with which to examine microalgal growth and lipid productivity, as was demonstrated previously (Burkert et al. 2004; Ghosh et al. 2015).

### Microalgal growth in photobioreactors

Given the difficulty in keeping outdoor photobioreactors sterilized, the cell count was used to assess the level of contamination, and the lack of a high number of contaminants was probably due to the alkalinity of the medium, as it was maintained at pH 9.0 during the growth period. The biomass productivity of the outdoor, vertical photobioreactor culture of *N. oleoabundans* was 1.75 g L^−1^. We found that medium with a glucose concentration of 7.5 g L^−1^ was ideal for achieving the highest cell biomass accumulation in a mixotrophic culture of *N. oleoabundans*.

This low biomass value may be due to FFAs and substances derived from the oxidation of unsaturated fatty acids. According to Sabia et al. (2015), these compounds are metabolites that have inhibitory effects on microalgae, and they influence the production of biomass in mixotrophic culture media. We found that the *N. oleoabundans* yield coefficient based on the glucose concentration was only 23 % when calculated using the highest biomass productivity (1.75 g L^−1^) divided by glucose concentration (7.5 g L^−1^). In fact, it should be less than 23 %, as CO_2_ production via photosynthesis also contributed to the cell biomass. Under mixotrophic conditions it was demonstrated that *N. oculata* CCMP 525, *D. salina* FACHB 435, and *C. sorokiniana* CCTCC M209220 had different ability to use glucose, which ranged from 27 ± 1 to 93 ± 6 % according to glucose concentrations in the medium (Wan et al. 2011).

The effects of carbon supplementation were also studied by Giovanardi et al. (2014), who observed that a lower concentration of glucose (2.5 g L^−1^) was optimal for boosting the cell density and lipid accumulation in the biomass of *N. oleoabundans* UTEX#1185. These authors also concluded that the growth of this microalga was limited when the glucose concentration was greater than 10.0 g L^−1^. Studies of different microalgae also have showed varied responses to glucose or sodium acetate. For example, maximum biomass production (2.01 g L^−1^) by *Phaeodactylum tricornutum* UTEX#640 was achieved at a glucose concentration of 5.0 g L^−1^, while a sodium acetate concentration of 4.1 g L^−1^ yielded 1.15 g L^−1^ of biomass (Garcia et al. 2005). For *Chlamydomonas reinhardtii*, it was shown that a higher sodium acetate concentration (10 g L^−1^) was needed to produce 2.15 g L^−1^ of dry biomass (Moon et al. 2013).

The specific growth rate (µ) of *N. oleoabundans* was 0.145 d^−1^, without any additional light besides an average solar radiation of 414.3 W/m^2^. This specific growth rate was lower than that reported by Kong et al. (2013) for *C. vulgaris* grown in medium containing glycerol and glucose at 30 °C and an illumination of 2500 lux. The lower growth rate in our study may have been due to the experimental conditions, in which the vertical photobioreactor containing 20 L of medium was placed outdoors, where there was a low light intensity and the minimum nighttime temperature was 21.5 °C. A significant finding by Li and co-workers (2014) was that mixotrophic green microalgae showed evidence of improved specific growth rates with increasing light intensities.

The protein content in *N. oleoabundans* biomass ranges from 44 (Morales-Sanchez et al. 2013) to 45 % (Gatenby et al. 2003), showing that the protein content depends upon the growth conditions and the N factor that is used in the calculations. By comparing mixotrophic and phototrophic conditions, Alkhamis and Qin 2015 showed that there was a 2.5-fold increase in the protein content of biomass when the marine microalga *Tisochrysis lutea* was grown under mixotrophic conditions, compared with that obtained during growth under phototrophic conditions.

We observed that the lipid content of *N. oleoabundans* cells was similar to that reported by Li et al. (2008), who also observed a 34 % lipid content for this strain. Growth of *N. oleoabundans* UTEX#1185 is highly promoted during the first week of mixotrophic cultivation, while photosynthetic pigments and lipids are over-produced during the following 3 weeks (Baldisserotto et al. 2014).

The ^1^H-NMR spectrum can be interpreted by comparing the ratio between the glycerol/methylene signals, as shown by Carneiro et al. (2005) but differently from these authors, that assign to the sum of the peak area the amount of four hydrogens to the glycerol comparing with all other integrated peaks, the integration was compared with α-carbonyl methylene hydrogens (2.2–2.35 ppm) that doesn’t vary regardless the amount of FFA. A ^1^H-NMR spectrum revealed that the FFA content of *N. oleoabundans* lipid fractions was 11.25 %. This value is lower than the values observed by other authors for different algal genera. When considering biofuel production from microalgal feedstocks, the FFA analysis is an important step, because although the extracted oils from microalgal biomass have been generally been show to contain high FFA contents of up to 19 % of dry biomass, the saponifiable lipids and resulting biodiesel represent only 1 % of the dry weight (Krohn et al. 2011). By comparing the growth of the marine microalga *T. lutea*, Alkhamis and Qin 2015 observed that the addition of 50 mM glycerol as an organic carbon source to a mixotrophic culture changed the fatty acid profile and increased the overall algal biomass production. Biomass storage conditions can result in lipid degradation, which results in the release of volatile organic acids and/or the formation of FFAs (Foree and McCarty 1970). Chen et al. (2012) concluded that the lipid composition of wet algal biomass is modified during storage, and that high amounts of FFAs are produced by triacylglycerol hydrolysis at temperatures above the freezing point.

In conclusion, the Box–Behnken design is an effective tool by which to optimize the concentrations of glucose and sodium acetate to maximize biomass, lipid, and protein productivity by *N. oleoabundans*. This study also showed that optimizing the concentrations of glucose and sodium acetate when growing *N. oleoabundans* under mixotrophic conditions in a scaled-up photobioreactor can be used to generate biomass that is rich in proteins and that also has a high lipid content, which makes it a great potential feedstock for biofuel production.
